# Twenty-Year Experience in the Diagnosis and Treatment of Infective Endocarditis

**DOI:** 10.1371/journal.pone.0134021

**Published:** 2015-07-31

**Authors:** Elżbieta Abramczuk, Janina Stępińska, Tomasz Hryniewiecki

**Affiliations:** Institute of Cardiology, Warsaw, Poland; University of Iowa Carver College of Medicine, UNITED STATES

## Abstract

**Aims:**

The aim of this study was to compare the etiology, clinical course, selected diagnostic methods and efficacy of the treatment used in patients with infective endocarditis (IE) in the nineteen eighties and nineties.

**Material and Methods:**

The study group comprised 300 patients with infective endocarditis hospitalized in the Institute of Cardiology in Warsaw in the following years: from 1982 to 1987 (150 patients: 75 successive patients with IE on the prosthetic valve and 75 successive patients with IE on the native valve), as well as from 1990 to 2003 (150 patients: 75 successive patients with IE on the prosthetic valve and 75 successive patients with IE on the native valve).

**Results:**

In the nineties, immunological symptoms, embolism formation and progressive heart failure were diagnosed decidedly more frequently. Early prosthetic valve endocarditis (PVE) (up to 60 days after operation) occurred significantly more frequently in the eighties. The quantity of negative blood cultures in PVE has not decreased, it is still observed in over 20% of cases. For 20 years the etiology of PVE has remained the same, the dominant pathogen remains *Staphylococcus*. The frequency of PVE caused by S*treptococci* has markedly reduced. In both the decades analyzed the etiology of native valve endocarditis (NVE) was similar. In the eighties *Streptococcus* was predominant. In successive years the number of infections caused by *Staphylococci* was the same as that caused by *Streptococci*.

**Conclusions:**

The incidence of early PVE decreased in the nineties. More patients were treated surgically with lesser peri-operative mortality. A lower incidence of infective endocarditis on prosthetic valves caused by streptococci may signify better prophylaxis against infective endocarditis. Infective endocarditis with sterile blood cultures continues to occur frequently.

## Introduction

Infective endocarditis (IE) is a relatively rare disease, it occurs in 1.5–7.0/100 000 of the population per year [1–3]. In the last 20 years changes in the epidemiology of IE have become evident. There has been a gradual increase in the frequency of occurrence in patients without predisposing heart disease. The most frequent risk factor for IE in patients with no previously seen structural changes in the heart is intra-venous drug abuse, chronic hemodialysis and central venous access [4, 5]. In these patients the disease involves the tricuspid valve, less frequently the mitral, contrary to those patients with known pathology [4, 6]. Despite modern methods of treatment: the availability of a wide range of antibiotics, appropriate qualification for cardiac surgery, infective endocarditis remains a serious illness with a high risk of death [7]. Mortality, according to various authors, in the case of native valve endocarditis (NVE) is from 7.6% to 20%, and in the case of prosthetic valve endocarditis (PVE)—from 13% to 56% [4, 8–14].

The criteria for diagnosing and treating IE have been being updated and discussed for many years. Already in 1646, Lazar Riviere described endocarditis, on the basis of an autopsy, when he found spherical vegetations in the left ventricle reminiscent of pleural tissue. In 1885, Osler reported the results of tests performed after the death of patients with endocarditis in the British Medical Journal [15]. From 1941, that is, when penicillin was discovered, treatment of IE with antibiotics was initiated, giving a chance of survival, but still over 50% of patients died from cardiac insufficiency secondary to the destruction of the heart valves involved. First reports of the surgical treatment of IE come from the nineteen sixties [16, 17]. In 1981, von Reyn et al. from Beth Israel Hospital in Boston proposed diagnostic criteria for IE based on the results of histopathology tests, clinical symptoms and blood cultures [18]. In succeeding years this classification was criticized, as echocardiography was not taken into account. New diagnostic criteria were proposed in 1994 by Durack et al. from Duke University Medical Centre in Durham, North Carolina. These criteria did take into account the possibility of using echocardiography to picture IE [19]. It was found that the sensitivity of trans-thoracic echocardiography (TTE) was 60%, and that of trans-esophageal echocardiography (TEE) was over 90% [20]. Currently echocardiography plays a fundamental role in the diagnosis of IE. It delivers a rapid and detailed picture of the damaged structures of the heart, giving cardiac surgeons the possibility of preparing appropriate surgical technique [21]. The most recent recommendations of the group of experts from the European Society of Cardiology (ESC), which appeared in the European Heart Journal in 2004, introduce new terminology concerning IE. It includes data concerning the activity of the process (active, recurring), the diagnosis (certain, probable and possible), the pathogenesis (IE on native valves, on prosthetic heart valves), the location (right heart, left heart) and the microbiology (cultures, serology, histology, PCR positive or negative) [21]. In recent years, automatic systems, allowing for the detection of the presence of bacteria in the blood and other body fluids earlier than in the classical method, have been introduced.

Despite the broad dissemination of the regulations for taking blood samples, the frequency of negative blood cultures still remains at 5–31% [22–26] and has stayed at the same level for years [27]. Negative blood cultures pose a significant problem in the diagnosis and treatment of IE. Therefore new diagnostic tests are being sought for IE. For example, the polymerase test is recommended, which reveals bacterial and fungal DNA [2, 3, 15, 19, 21, 28–30]. According to new recommendations, in the case of negative blood cultures, serological tests should be carried out for *Chlamydia*, *Coxiella burnetii*, *Brucella*, *Legionella* and to detect fungi. Obtaining a positive blood culture or a positive serological reaction permits the correct diagnosis of IE and gives the opportunity to institute the appropriate pharmacotherapy.

The aim of this study was to compare the etiology, clinical course, selected diagnostic methods and efficacy of the treatment used in patients with infective endocarditis in the nineteen eighties and nineties.

## Materials and Methods

The study group comprised 300 patients with infective endocarditis hospitalized in the Institute of Cardiology in Warsaw in the following years:

- from 1982 to 1987

150 patients: 75 successive patients with IE on the prosthetic valve and 75 successive patients with IE on the native valve, as well as

- from 1990 to 2003

150 patients: 75 successive patients with IE on the prosthetic valve and 75 successive patients with IE on the native valve.

Diagnosis of infective endocarditis in the eighties was based on clinical examination, laboratory tests (erythrocyte sedimentation rate—ESR, leucocytosis, etc.), blood cultures and echocardiography (low quality). In the nineties the diagnosis of IE was based on clinical examination, laboratory tests (ESR, CRP, leucocytosis, etc.), blood cultures and high quality echocardiography (trans- thoracic and trans-esophageal). The Duke University diagnostic criteria were introduced.

In the eighties a lot of blood cultures were performed 6–8 times a day. In the nineties, reduced the number of cultures to 3 times every hour. Serologic tests were not applied.

The following parameters were compared:

- presence of pyrexia, immunological symptoms, embolism formation and cardiac insufficiency
- presence of early IE,—results of blood cultures and valves
- etiology,—medical or surgical treatment
- results of treatment (deaths—in-hospital mortality, recurrences)

The results were analyzed using the Chi-squared test. Where the numbers were less than 5, the accurate Fischer test was used. A level of significance of p < 0.05 was accepted for verification of the hypotheses.

The study was reviewed and approved by an Ethics Committee of Institute of Cardiology (Warsaw, Poland) before the study began. The participants provided their written informed consent to participate in this study.

## Results and Discussion

### Diagnosis of IE

Over the years the localization of PVE has not changed. It affects the mitral and the aortic openings to the same degree. Pyrexia is a permanent symptom accompanying infection in over 90% of cases. In the nineties, immunological symptoms, embolism formation and progressive heart failure were diagnosed decidedly more frequently (Table 1).

**Table 1:** Prosthetic and native valve endocarditis.

	PVE			NVE		
Years	1982–1987	1990–2003	p	1982–1987	1990–2003	p
**N**	75	75		75	75	
**Mitral valve**	31 (41.3%)	27 (36%)	NS	15 (20%)	15 (20%)	NS
**Aortic valve**	28 (37.3%)	28 (37.3)	NS	36 (48%)	38 (50.7%)	NS
**Mitral and aortic valves**	16 (21.3%)	17 (22.7%)	NS	24 (32%)	19 (25.3%)	NS
**Tricuspid valve**	0	3 (4%)	NS	0	3 (4%)	NS
**Pyrexia**	71 (94.7%)	71 (94.7%)	NS	72 (96%)	72 (96%)	NS
**Immunological symptoms**	2 (2.7%)	20 (26.7%)	< 0.05	5 (6,7%)	9 (12%)	NS
**Embolism**	13 (17.4%)	19 (25.3%)	< 0.05	18 (24%)	8 (10.7%)	NS
**Heart failure**	24 (32%)	48 (64%)	< 0.05	42 (56%)	39 (52%)	NS

PVE—prosthetic valve endocarditis; NVE—native valve endocarditis; N—number; p—p value; NS—non significant.

Early PVE (up to 60 days after operation) occurred significantly more frequently in the eighties. The quantity of negative blood cultures in PVE has not decreased, it is still observed in over 20% of cases. For 20 years the etiology of PVE has remained the same, the dominant pathogen remains *Staphylococcus*. The frequency of PVE caused by S*treptococci* has markedly reduced (Table 2).

**Table 2:** Prosthetic and native valve endocarditis: microbiological tests’ results.

	PVE			NVE		
Years	1982–1987	1990–2003	p	1982–1987	1990–2003	p
**N**	75	75		75	75	
**Early PVE**	48 (64%)	17 (22.6%)	< 0.001			NS
**Positive blood cultures**	57 (76%)	55 (73%)	NS	47 (62.5%)	43 (57%)	NS
**Staphylococci**	34	26	NS	17	17	NS
**Streptococci**	13	4	< 0.05	22	16	NS
***Propionibacerium***	0	11	NS	0	1	NS
**Others**	10	14	NS	8	9	NS
**Positive valve cultures**	14	9	NS	15	15	NS

PVE—prosthetic valve endocarditis; NVE—native valve endocarditis; N—number; p—p value; NS—non significant.

In both the decades analyzed the etiology of NVE was similar. In the eighties *Streptococcus* was predominant. In successive years the number of infections caused by *Staphylococci* was the same as that caused by *Streptococci*. A high percentage of negative blood cultures continued to be registered—in over 30% of cases (Table 2). There was no isolation of HACEK (*Haemophilus*, *Aggregatibacter*, *Cardiobacterium*, *Eikenella*, *Kingella*) organisms.

### Treatment of IE

In the nineties patients with PVE were treated surgically significantly more frequently. In-hospital mortality in this group of patients decreased clearly in the nineties. Medical treatment was dominant in the eighties (Table 3).

**Table 3:** Prosthetic and native valve endocarditis—treatment and outomes.

	PVE			NVE		
Years	1982–1987	1990–2003	p	1982–1987	1990–2003	p
**N**	75	75		75	75	
**Medical**	38 (50.6%)	24 (32%)	< 0.05	17 (22.7%)	8 (10.7%)	NS
**Death**	10 (26%)	4 (16,6%)	NS	6 (35%)	1 (12.5%)	NS
**Surgical**	37 (49.4%)	51 (68%)	< 0.05	58 (77.3%)	67 (89.3%)	NS
**Death**	19 (51.3%)	9 (17.6%)	< 0.01	8 (13.7%)	2 (2.9%)	< 0.01

PVE—prosthetic valve endocarditis; NVE—native valve endocarditis; N—number; p—p value; NS—non significant.

The majority of patients with NVE were treated surgically, both in the eighties and the nineties. A clear reduction in the in-hospital mortality of this group of patients can be seen (Table 3).

Despite a continuously decreasing number of cases of rheumatic fever, previously regarded as being the main factor predisposing to IE, the incidence of IE is not falling. Several factors exert an influence on this: new risk groups, new strains of antibiotic resistant bacteria, and the high detectability of IE, particularly thanks to echocardiography [21]. Infective endocarditis still carries a high mortality, particularly when a prosthetic valve is involved [13, 31–34].

The etiology of IE has been changing over successive years, and it also differs in various countries. In the 70s and 80s *Streptococcus viridans* was dominant in the etiology of IE, this was presented in studies by, among others, Delahay et al. from France [25], as well as Vlesis from the USA [10]. In succeeding years, in line with the increase in use of prosthetic valve implants *Streptococcus viridans* became the main pathogenic factor only in NVE [8], and coagulase-negative *Staphylococcus* in PVE [32, 35, 36]. In Japan, according to the study of Ako from 1980–1999 *Streptococcus viridans* continues to dominate in the etiology of IE [37]. In Israel, where there is a high incidence of rheumatic fever, cultures from patients with NVE mainly grow *Streptococcus viridans*, and in PVE *Enterococcus* [38]. Observations of many authors from the UK [13, 23], Spain [12], Switzerland [7], Germany [39], as well as Italy [40] and Canada [41] show a current clear rise in the participation of *Staphylococcus* in the etiology of IE.

In materials presented in 1982–2003, on the basis of the results of blood cultures and from the valves themselves, it has been observed that staphylococci are grown more and more frequently in NVE. These results differ from those of previous years: at that time, similarly as in other countries, the most common etiological factor was *Streptococcus viridans*. In interpreting this result the over 30% of negative blood cultures should be borne in mind. Such a high percentage of negative blood cultures, associated most probably with antibiotic therapy administered prior to referral to the reference centre and taking of blood cultures, may falsify the correct evaluation of etiological factors. On the basis of the results obtained, it should be deemed that the etiology of NVE in Poland is changing: the number of cases of NVE caused by staphylococci is increasing. In the eighties was found five cases of *Staphylococcus aureus* among staphylococcal IE, and in the nineties ten cases. In the eighties was found five cases of *Staphylococcus aureus* among staphylococcal IE, and in the nineties ten cases.

Karchmer also notes the role of coagulase-negative staphylococci in the etiology of NVE. According to Karchmer, these strains differ from those seen in PVE in 70% sensitivity to anti-staphylococcal penicillins [42]. From analysis of the data of patients with PVE it transpires that the main pathogenic factor is *Staphylococcus epidermidis*. This etiology has not changed for almost 20 years. The results of the study presented do not differ from the results of other authors in Europe or Canada [7, 12, 13, 23, 39, 40, 41, 43]. In recent years Selton et al. showed staphylococci were the most common causal agents of IE [44]. In subsequent years, there was an increase the incidence of staphylococci in the etiology of IE [44–48].

Since the nineties, a significant decrease in early mortality in IE has been noted. In France early mortality in NVE has decreased from 21.6% to 16.6% [4], in Spain from 19% to 12% [8]. In Great Britain hospital mortality in NVE is 7.6%, and in PVE 16.6% [13]. In the USA hospital mortality in PVE has decreased from 20% to 10% [14]. According to a publication by Delay et al. from Canada, early mortality in NVE was 8%, and in PVE there were no deaths. This is the only publication in which the authors report a lack of early mortality in the group of patients most difficult to treat with PVE. Perhaps it is a result of work organization—transferring patients early after their operations to other centers or premature discharge from hospital. The very high annual mortality in this group—26%, is in favor of this [41]. In the group studied early mortality in NVE decreased from 18% to 4%, and in PVE from 38% to 17%. These values are comparable to world results.

Mortality is still quite high particularly in the group with PVE, however, compared to the data of 10–15 years ago, it is significantly lower. Such high efficacy in the treatment of IE has been achieved thanks to modern antibiotic therapy, earlier qualification for surgical treatment, the experience of cardiac surgeons and professional peri-surgical care. Still in the eighties, treatment with II generation cepaholosporins in association with netilmicin was introduced. Netilmicin was used due to its potentially lesser ototoxicity and nephrotoxicity to gentamicin. In complicated streptococcal infections and staphylococcal infection with meticillin-resistant *Staphylococcus epidermidis* (MRSE) strains, vancomicin was already being used at that time. Infective endocarditis caused by Gram-negative flora or anaerobes was treated with imipenem with cilastatin. Teicoplanin was introduced into the treatment of IE caused by enterococci and MRSE strains of staphylococci already in 1996. This anti-microbial therapy preceded current European standards by several years [21].

Cardio-surgical treatment, particularly early qualification for surgical treatment, is the basic factor lowering the risk of death [7, 10, 11]. In the publication of Ivert et al. from 1984 concerning 53 patients with PVE, mortality in the group treated medically was 70%, and in the group treated surgically 24% [33]. In contemporary literature mortality in the group of re-operated patients during the course of PVE amounts to 10–19% [13, 14, 34].

Studies to date indicate that the prognosis for patients suffering from PVE is worse, that both pharmacological and surgical treatment of this group of patients is more difficult. Patients with NVE are younger, they usually have a severe and reversible form of cardiac insufficiency, indeed they often return to a normal lifestyle and professional activity after recovery from IE. However, patients with PVE have a background of many years of a progressive, acquired heart defect leading to irreversible hemodynamic consequences. After recovering from infective endocarditis they continue to experience symptoms of cardiac insufficiency requiring permanent pharmacotherapy. The difference in the course of infective endocarditis also stems from the difference in construction of the native and prosthetic heart valve. The inflammatory process on the native valve damages the cusps, the sub-cusp apparatus and creates abscesses in the endocardium. After the implantation of prosthetic valves the conditions for the development of vegetation and the formation of infected thrombi are better around the valve’s ring. The inflammatory process initially occurs near the stitches of the prosthetic valve, and peri-valvular abscesses form, often the infection also involves the neighboring endothelium or seeps into the chamber’s muscle [21]. The long-term results of combined therapy of IE: targeted anti-microbial therapy and cardio-surgical therapy continue to improve. Average 5-year survival after cured NVA fluctuates among researchers between 75% and 88% [10, 12, 41]. In PVE 5-year survival is 59% to 82% [12, 41, 43, 49].

## Conclusions

1. The incidence of early PVE decreased in the nineties.
2. More patients were treated surgically with lesser peri-operative mortality.
3. A lower incidence of infective endocarditis on prosthetic valves caused by streptococci may signify better prophylaxis against infective endocarditis.
4. Infective endocarditis with sterile blood cultures continues to occur frequently.
